# Characterization of the Treg Response in the Hepatitis B Virus Hydrodynamic Injection Mouse Model

**DOI:** 10.1371/journal.pone.0151717

**Published:** 2016-03-17

**Authors:** Kirsten K. Dietze, Simone Schimmer, Freya Kretzmer, Junzhong Wang, Yong Lin, Xuan Huang, Weimin Wu, Baoju Wang, Mengji Lu, Ulf Dittmer, Dongliang Yang, Jia Liu

**Affiliations:** 1 Institute for Virology, University Hospital of Essen, University of Duisburg-Essen, Essen, Germany; 2 Department of Infectious Diseases, Union Hospital, Tongji Medical College, Huazhong University of Science and Technology, Wuhan, China; Institut Pasteur, FRANCE

## Abstract

Regulatory T cells (Tregs) play an important role in counter-regulating effector T cell responses in many infectious diseases. However, they can also contribute to the development of T cell dysfunction and pathogen persistence in chronic infections. Tregs have been reported to suppress virus-specific T cell responses in hepatitis B virus (HBV) infection of human patients as well as in HBV animal models. However, the phenotype and expansion of Tregs has so far only been investigated in other infections, but not in HBV. We therefore performed hydrodynamic injections of HBV plasmids into mice and analyzed the Treg response in the spleen and liver. Absolute Treg numbers significantly increased in the liver but not the spleen after HBV injection. The cells were natural Tregs that surprisingly did not show any activation or proliferation in response to the infection. However, they were able to suppress effector T cell responses, as selective depletion of Tregs significantly increased HBV-specific CD8+ T cell responses and accelerated viral antigen clearance. The data implies that natural Tregs infiltrate the liver in HBV infection without further activation or expansion but are still able to interfere with T cell mediated viral clearance.

## Introduction

Chronic hepatitis B virus (HBV) infection continues to be one of the major public health problems worldwide [1, 2]. Two billion people have been infected with HBV, of whom about 248 million developed chronic infection [3]. Every year, approximately one million of these individuals will die from HBV-associated liver diseases such as cirrhosis and hepatocellular carcinoma (HCC) [1, 2]. The host immune response determines whether acute HBV infection will progress to resolution or chronicity. A strong, multi-specific T cell response to HBV antigens is associated with the clearance of hepatitis B [4]. Depletion of CD8+ T cells in chimpanzees during acute HBV infection results in the persistence of viremia [5], indicating that virus-specific T cells play a pivotal role in HBV control. In contrast, weak or undetectable HBV-specific CD8+ T cell responses correlate with HBV persistence [6–9].

A number of reports have suggested that CD4+Foxp3+ regulatory T cells (Tregs) play a significant role in suppressing T cell responses during viral infections [10, 11]. The mechanism of viral immune escape and persistence by induction of Tregs was first described in the Friend virus (FV) mouse model [12]. During acute FV infection, an expanding population of Tregs suppresses the antiviral function of virus-specific CD8+ T cells [13–15], which results in T cell exhaustion, contributing to the establishment of a chronic infection [16]. The depletion of Tregs during the acute phase of FV infection resulted in enhanced effector T cell function and decreased viral loads [15, 17]. A transient depletion of Tregs in an established chronic FV infection partly improved anti-viral immune responses by reactivating previously suppressed and functionally exhausted CD8+ T cells. Reactivated CD8+ T cells were subsequently able to significantly reduce chronic viral set points [16, 18].

The phenotype of the virus-induced Tregs has also been analyzed in great detail in the FV model. Tregs are divided into two subsets based on their origin: natural Treg (nTreg) that develop in the thymus and inducible Treg (iTreg) that arise by the induction of FoxP3 in conventional CD4+FoxP3− T cells in the periphery [19–21]. In FV infection only very few iTregs can be found, but nTregs become activated and massively expand in sides of virus replication [22–25]. Recently, we demonstrated that FV infection induced disproportionately greater expansion of nTregs expressing the TCR Vβ5 chain compared with nTregs expressing other Vβ-chains [26]. Investigations revealed that the polyclonal population of non-Vβ5+ Tregs likely recognizes self antigens and are expanded by IL-2 signaling [26, 27], whereas the Vβ5+ Treg population responds to an endogenous retroviral superantigen (MMTV-9 Sag) and tumor necrosis factor receptor (TNFR) 2 signaling [28]. The TNFR2 signal comes from activated effector CD8+ T cells that express membrane-bound TNF-α. [28]. The infection induced activation of Tregs leads to a massive up-regulation of activation markers (e.g. Helios, TNFR2), proliferation, and differentiation.

Accumulating evidence also indicates that Tregs play an important role in down-regulating HBV-specific effector T cell responses in HBV patients [29–31]. For example, in a study which enrolled 50 chronic hepatitis B (CHB) patients, 23 healthy controls (HC), and 9 individuals with a resolved HBV infection, a significantly higher percentage of Tregs was observed in PBMCs of CHB patients compared with HC and resolvers [29]. An association of increased peripheral Treg numbers with persistence of HBV infection was also confirmed in another study which enrolled 79 CHB patients, 26 asymptomatic carriers, 12 acute hepatitis B infections, and 20 HC [32]. Increased Tregs frequencies in liver-infiltrating lymphocytes were also observed in CHB patients with severe hepatitis compared to HC [30]. Tregs from CHB patients produced interleukin (IL)-10 but little or no interferon (IFN)-gamma upon TCR stimulation [32]. Importantly, Tregs of CHB patients were capable of suppressing the function of effector T cells in vitro. Depletion of Tregs in culture enhanced the proliferation of HBV-specific effector T cells and secretion of interferon-γ (IFN- γ) [29, 30]. In a mouse model utilizing HBV-transgenic Adeno-associated virus vectors, Treg depletion also increases early CD8+ T effector functions and immune control of acute HBV infection in vivo [33]. Furthermore, Tregs controlled the recruitment of innate immune cells such as macrophages and dendritic cells to the HBV infected liver. As a consequence, Tregs significantly delayed clearance of HBV from blood and infected hepatocytes [33].

Despite these intensive studies on the role of Tregs in HBV infection, the phenotype and activation, proliferation, and differentiation status of Tregs in HBV infected humans or mice has never been characterized in detail. Therefore, in the current study, we provide a characteristic analysis of HBV induced Tregs and their effect on virus specific CD8+ T cell response by using the HBV hydrodynamic injection mouse model.

## Materials and Methods

### Mice

Inbred C57BL/6 wild type (WT) mice and DEREG mice [34] were maintained under pathogen free conditions at the Institute for Virology, University Hospital Essen. All mice were males of 6–8 weeks of age at the beginning of the experiments and were sacrificed by cervical dislocation at the end of the experiments. Animal experiments were performed in strict accordance with the German regulations of the Society for Laboratory Animal Science (GV-SOLAS) and the European Health Law of the Federation of Laboratory Animal Science Associations (FELASA). The protocol was approved by the North Rhine-Westphalia State Agency for Nature, Environment and Consumer Protection (LANUV), which is the animal ethics committee of the local government. All efforts were made to minimize suffering.

### Hydrodynamic injection in mice

A replication-competent HBV clone pSM2 harboring a head-to-tail tandem dimeric HBV genome (GenBank accession number: V01460) was provided by Dr. Hans Will (Heinrich-Pette-Institute, Hamburg, Germany) and used previously in our lab [35]. Male mice at 6–8 weeks of age were hydrodynamically injected (HI) with 10 μg pSM2 in a volume of phosphate-buffered saline (PBS) solution equivalent to 0.1 ml/g of the mouse body weight through tail veins within 8 seconds [36, 37].

### Isolation of lymphocytes from the spleen and liver

Preparation of single-cell suspensions of murine splenocytes was performed according to a protocol described previously [38]. Mouse intrahepatic lymphocytes (IHLs) were isolated as described previously [39]. In brief, the mouse liver was perfused immediately with 10 ml PBS after sacrifice. After perfusion, the liver was homogenized and digested with enzyme solution containing 0.05% collagenase type IV (Sigma-Aldrich), 0.002% DNAase I (Sigma-Aldrich), and 10% fetal bovine serum for 30 minutes. The pellet after digestion was resuspended in 40% Percoll and centrifuged at 1000 g without break off. After removing the debris and hepatocytes on the top layer, IHLs in the pellet were collected, washed, and subjected to further analysis.

### Cell surface and intracellular staining by flow cytometry

Surface and intracellular staining were performed as described previously [18, 40]. The gating strategy for Tregs is shown in S1 Fig. For staining CD8^+^ T cells specific to the K^b^-HBV Cor_93-100_ (MGLKFRQL), recombinant soluble dimeric mouse H-2K[b]:Ig fusion protein (DimerX I, BD Bioscience) were loaded with HBV Cor_93-100_ for over night, and then used to stain mouse lymphocytes according to the technical instruction. The cells were incubated with CD16/CD32 rat anti-mouse antibody (clone 2.4G2; BD Pharmingen) to block FcRs. After washing, dimer staining was performed by incubation of dimer and cells for 1.5 hours at 4°C. The cells were washed and incubated with anti-IgG1 antibody (clone 85.1; eBioscience) for 30 minutes at 4°C. Data were acquired on a LSR II flow cytometer (Becton Dickinson) from 350,000–500,000 lymphocyte-gated events per sample. Analyses were done using FACSDiva software (Becton Dickinson) and FlowJo software (Treestar).

### Detection of serum HBV DNA

Serum HBV DNA was extracted by using QiAamp DNA Blood Mini kit (Qiagen) and quantified by real time PCR using a Platinum SYBR green Kit (Invitrogen) as described [35, 41].

### Treg depletion

To deplete Tregs, HBV HI mice were injected intraperitoneally with diphtheria toxin (Merck, Darmstadt, Germany), diluted in endotoxin-free PBS. 0.5 μg DT was inoculated every second day for 3 times. The treatment depleted over 97% of the CD4+ eGFP+ T cells in all investigated organs of DEREG mice.

### Statistical analysis

Statistical data were derived by using the GraphPad Prism software (GraphPad Software). Data were analyzed using oneway ANOVA and Newman-Keuls multiple comparison test, non-parametric one-way ANOVA and Dunn’s multiple comparison test or Student’s t-test.

## Results

### Hydrodynamic injection of HBV increases absolute numbers of Tregs in the liver but not spleen of mice

**Fig 1:**
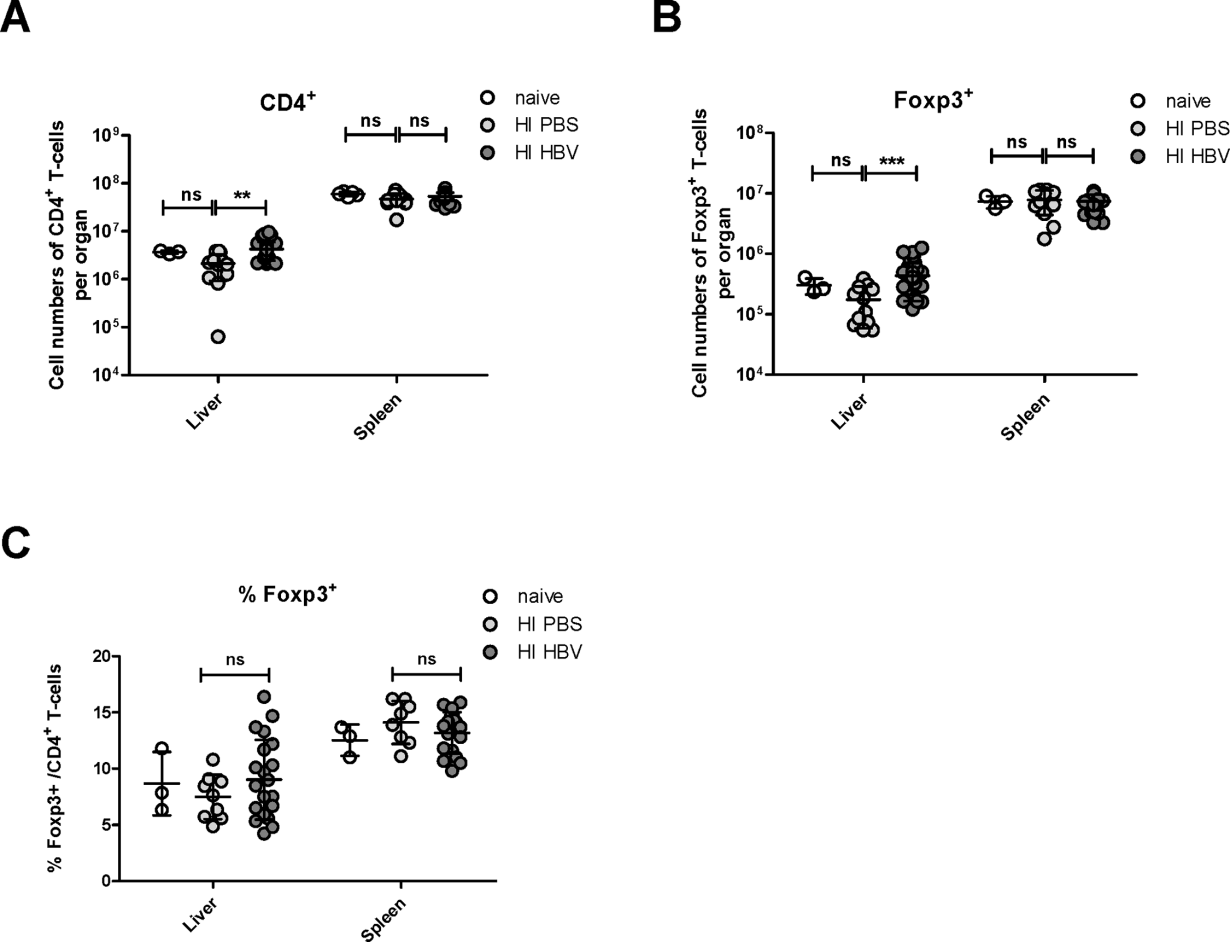
Quantification of Tregs in the liver and spleen of HBV-replicating mice. C57BL/6 mice were hydrodynamically injected with PBS or pSM2 plasmid. Flow cytometry was used to quantify CD4+ T cells and CD4+Foxp3+ Tregs in the liver and spleen 21 days after injection. (A) Absolute numbers of CD4+ T cells per organ were counted in the liver and spleen. (B) Absolute numbers of CD4+ Foxp3+ Tregs per organ were counted in the liver and spleen. (C) Treg percentages of total CD4+ T cells were calculated in the liver and spleen. Each dot represents an individual mouse. Data were pooled from 3 to 5 independent experiments with similar results. Statistical data were derived by using the GraphPad Prism software (GraphPad Software). All data sets were first tested for normality using the D’Agostino and Pearson omnibus normality test. Data was then assessed using an unpaired Student’s T-test. Statistically significant differences are indicated by asterisks (**p < 0.01, ***p < 0.001).

It has previously been shown that HBV infection can increase Treg numbers in the liver [30, 33]. To analyze whether hydrodynamic injection (HI) of HBV plasmids into mice also induces Treg responses we quantified Tregs in the liver and spleen of C57B/L6 mice injected with the plasmid pSM2 [35]. Similar to the previous report using pAAV-HBV1.3 [42], the HI of pSM2 leads to a typical acute HBV replication course in C57B/L6 mice. 21 days post HI absolute numbers and frequencies of total CD4+ T cells and Tregs were determined. Fig 1A shows that HI of HBV plasmids resulted in a significant increase of total CD4+ T cells in the liver but not the spleen compared to control mice that received a HI with PBS. Although an individual mouse in HI PBS group showed exceptional low numbers of liver CD4+ T cells, the difference between the two groups even stands when we removed this outlier from the analysis (data not shown). Similar results were found for total numbers of Tregs. Significantly more CD4+ Foxp3+ cells were detected in the liver but not the spleen of mice receiving HBV plasmids in comparison to the PBS control group (Fig 1B). Interestingly, the difference between the groups was lost when Treg percentages of total CD4+ T cells were calculated (Fig 1C). These results suggest that total CD4+ T cells including 5–15% Tregs infiltrated the liver after HI of HBV but no preferential infiltration or expansion of Tregs took place. However, it was still possible that the functional activity of liver infiltrating Tregs was affected upon HBV HI.

### The phenotype of liver infiltrating Tregs after HBV HI

**Fig 2:**
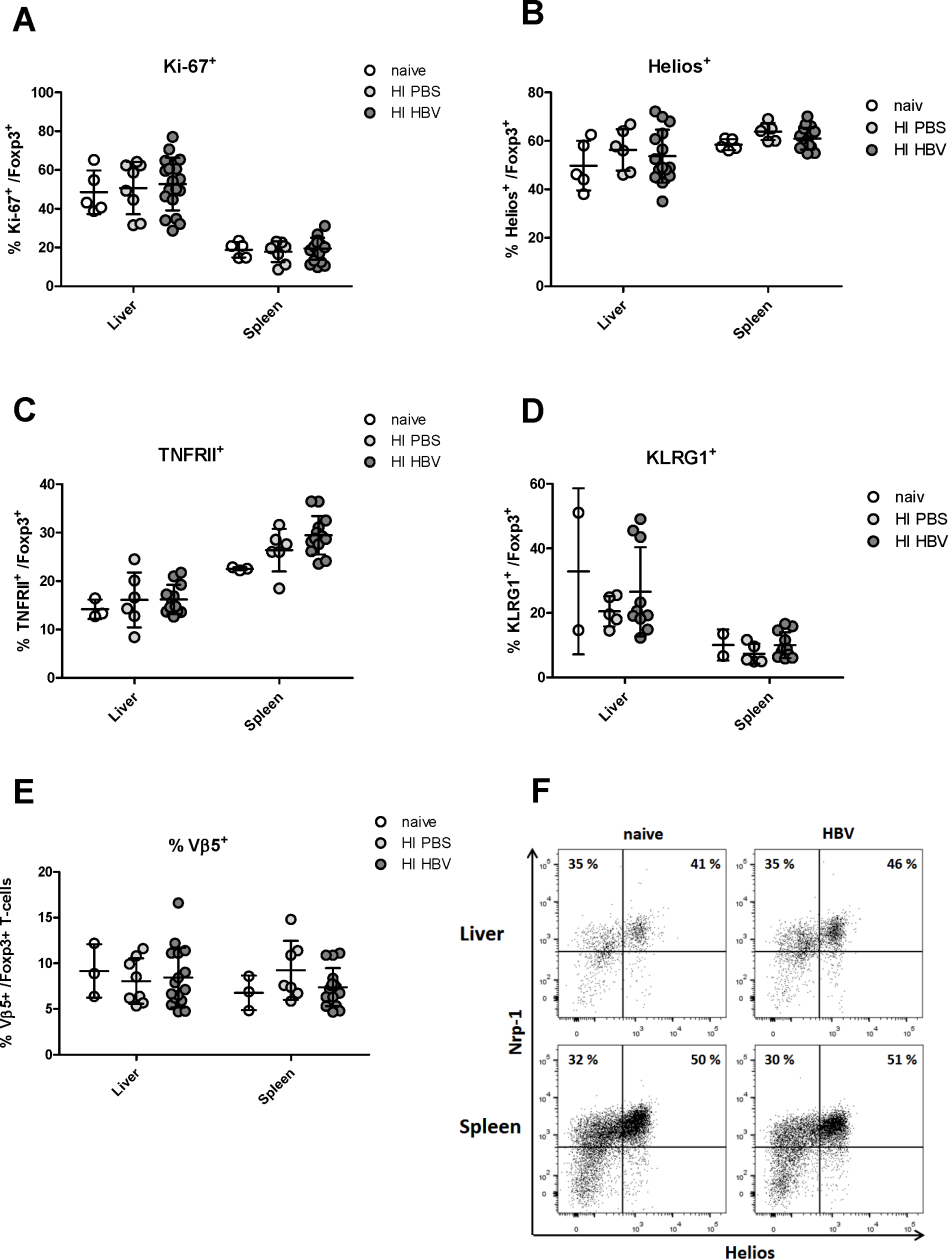
Phenotype analysis of Tregs in the liver and spleen of HBV-replicating mice. C57BL/6 mice were hydrodynamically injected with PBS or pSM2 plasmid. Flow cytometry was used to quantify activation and differentiation markers Tregs in the liver and spleen 21 days after injection. The frequencies of CD4+ Foxp3+ Treg populations in the liver and spleen expressing (A) Ki67+ cells, (B) Helios+ cells, (C) TNFR2+ cells, (D) KLRG1+ cells, and (E) Vß5+ cells were enumerated and expressed as a percentage of the Treg population. Each dot represents an individual mouse. Data were pooled from 3–5 independent experiments with similar results. (F) Representative scatter plots of Neuropilin-1 against Helios are shown from a naive mouse and a HBV-replicating mouse. Cells were gated on CD4+ Foxp3+ Tregs.

We have previously reported that the infection of mice with FV results in a massive redistribution, activation and proliferation of Tregs [13–15, 26, 28]. This was also shown for several other virus infections, including LCMV [43]. We were therefore interested in whether HBV HI also activates Tregs in mice, which had not been studied in previous reports on Tregs in HBV mouse models [33]. First, Treg proliferation was analyzed in the liver and spleen using the proliferation marker Ki-67. No difference was found in percentages of Ki-67+ cells among the CD4+ Foxp3+ Tregs in both organs of mice receiving HBV plasmids or PBS (Fig 2A). The frequencies of proliferating Tregs in both groups were identical with that of non-treated naive mice. Similar findings were made when activation of Tregs was analyzed using the two well-established Treg activation markers Helios and TNFR2 [22, 24]. HBV HI did not result in increased percentages of Tregs expressing theses activation markers in the liver or spleen of mice (Fig 2B and 2C). Also differentiation of Tregs, as measured by KLRG1 expression, was not induced by HI of HBV (Fig 2D). This was in clear contrast to several infections of mice with other viruses [25] (55 and 31). Beside the strong activation, proliferation, and differentiation of Tregs found in mice infected with the FV, this infection also induces a disproportional expansion of Tregs expressing the Vß5 chain of the T cell receptor [28]. We therefore analyzed the proportion of Vß5+ Tregs in HBV HI mice. Not surprisingly, no disproportional expansion of the Vß5+ Tregs was found in the liver or spleen after HBV HI compared to non-treated mice or mice receiving PBS (Fig 2E). To further characterize the Treg subsets in HBV HI mice, we examined the expression of Neuropilin-1, which is considered the best marker that distinguishes nTreg from iTreg [44], on Tregs in the liver and spleen. Again, we did not observe a significant change of Neuropilin-1 expression on both activated (Helios+) or non-activated (Helios-) Tregs after HBV HI (Fig 2F). We also did not observe any significant change on CD25 expression of Tregs after HBV HI (data not shown). Taken together, HBV HI results in an infiltration of CD4+ T cells including nTregs into the liver, but not to a further activation or proliferation of these cells.

### nTregs in the liver suppress HBV-specific CD8+ T cell responses

**Fig 3:**
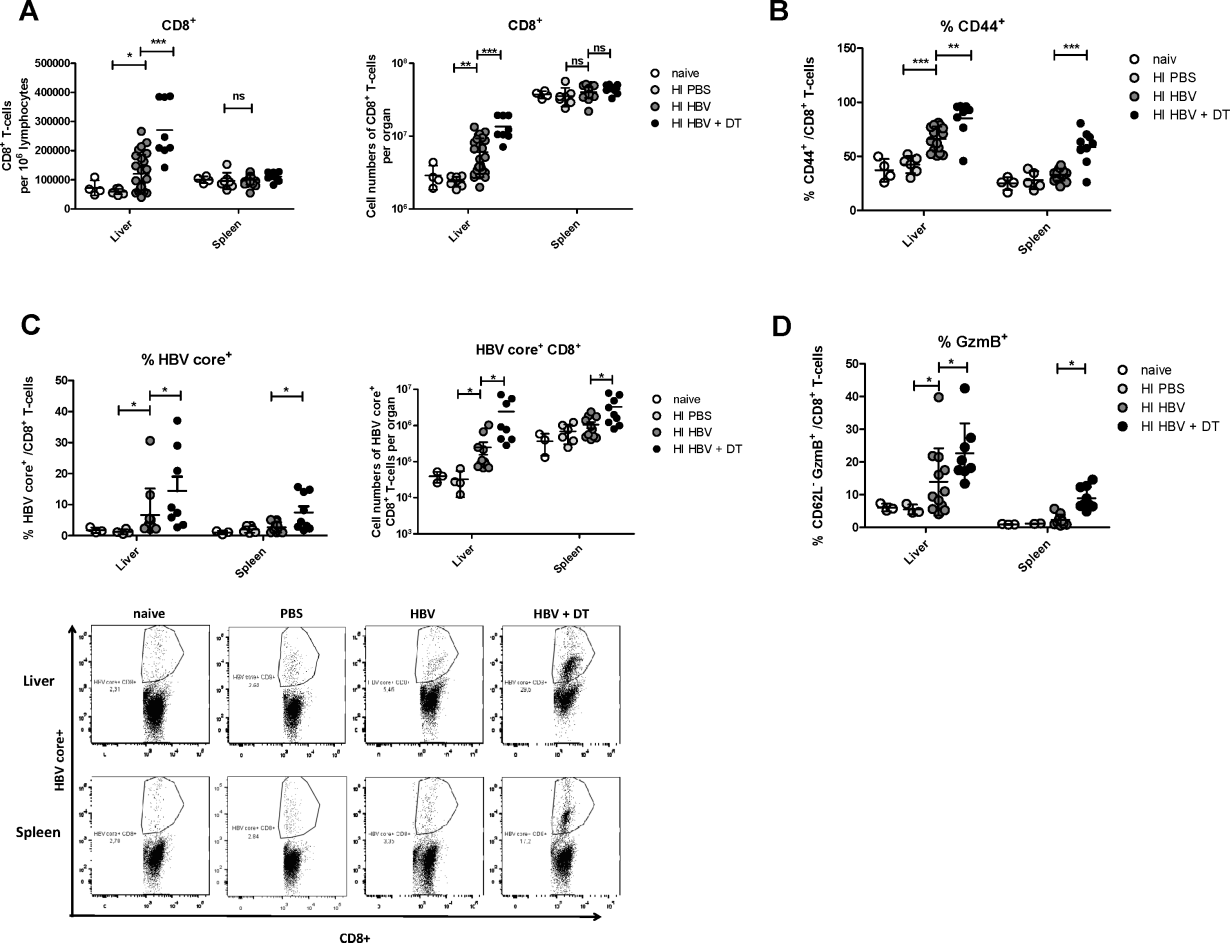
Impact of Treg depletion on CD8+ T cell responses in HBV-replicating mice. DEREG mice were hydrodynamically injected with PBS or pSM2 plasmid and were treated with DT to deplete Foxp3+ Treg on day 10, 12 and 14 after HI. Mice were sacrificed on 21dpi for CD8+ T cell response analysis by flow cytometry. (A) Absolute numbers of CD8+ T cells per 1 million lymphocytes and per organ were counted in the liver and spleen. (B) Activation markers CD44 expression in intrahepatic and splenic CD8^+^ T cells was measured. The frequencies of virus-specific class I dimer+ (C) and CD62L- granzyme B+ (D) CD8+ T cells in the liver and spleen were measured. Each dot represents an individual mouse. Data were pooled from 3–6 independent experiments with similar results. Statistical analysis and data presentation was performed using GraphPad Prism software. All data sets were first tested for normality using the D’Agostino and Pearson omnibus normality test. Data was then assessed using either parametric one-way analysis of variance (ANOVA). The statistical significances were calculated using the Tukey’s Multiple Comparison assessment methods. When only two groups were analyzed, data was tested using a parametric Student’s T-test. Statistically significant differences are indicated by asterisks (*p < 0.05, **p < 0.01, ***p < 0.001).

**Fig 4:**
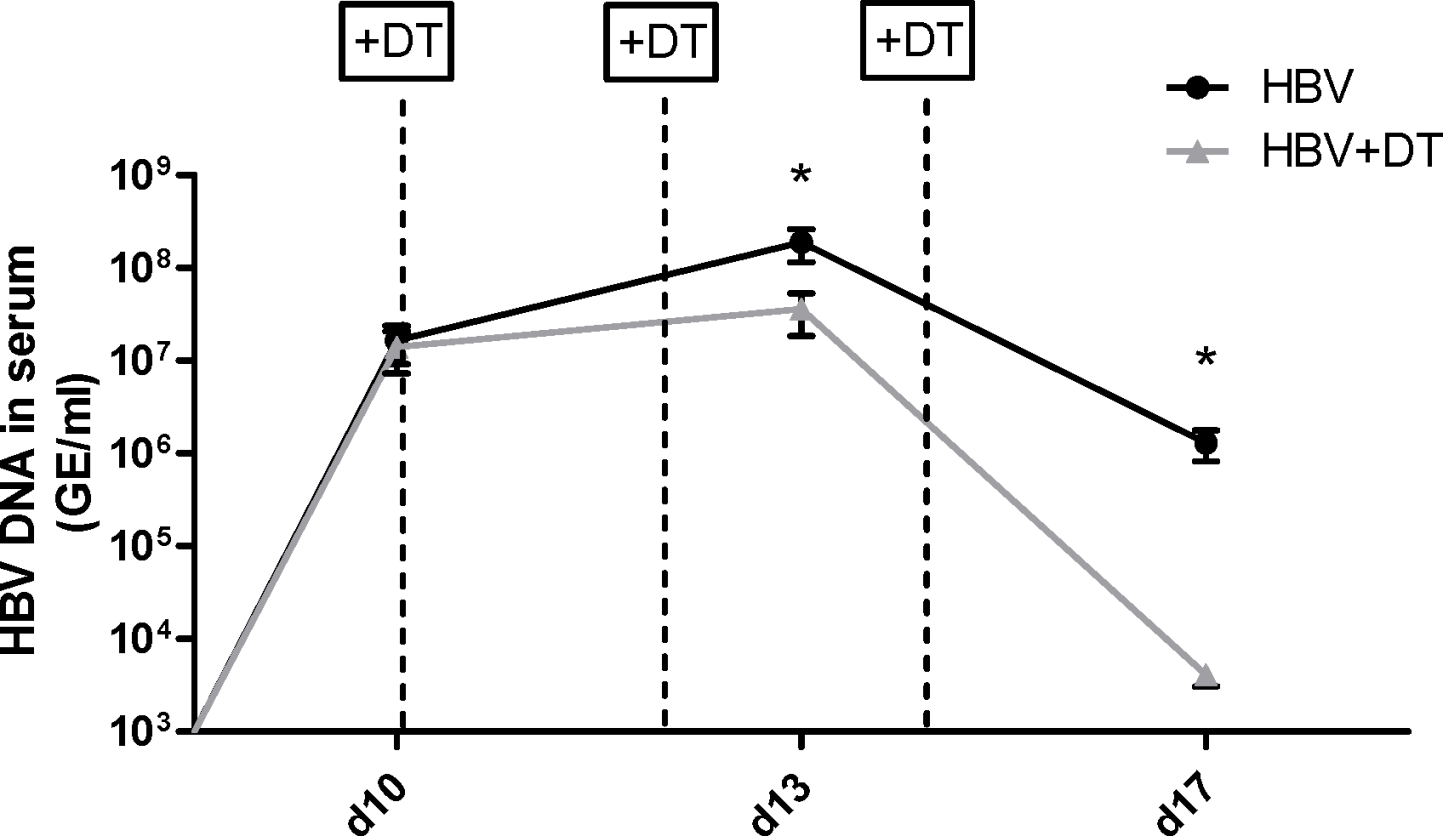
Impact of Treg depletion on HBV viral load in mice. DEREG mice were hydrodynamically injected with PBS or pSM2 plasmid and were treated with DT to deplete Foxp3+ Treg on day 10, 12 and 14 after HI. Mice were bled at indicated time points and HBV DNA levels in the serum were quantified by realtime PCR. Statistical data were derived by using the GraphPad Prism software (GraphPad Software). Data set was first tested for normality using the D’Agostino and Pearson omnibus normality test. Data was then assessed using an unpaired Student’s T-test. Statistically significant differences are indicated by asterisks (*p < 0.05)

To test whether or not the nTregs that infiltrate the liver without any significant virus-induced activation after HBV HI could still influence the CD8+ T cell responses to HBV, we analyzed numbers and functions of CD8+ T cells in the liver and spleen after Treg depletion. We took advantage of the DEREG mouse model [34] that enabled us to selectively deplete Tregs during HBV HI. DT treatment could result in depletion of about 81% eGFP+ Tregs in the liver in this mouse model (data not shown). Fig 3A shows that the absolute numbers of CD8+ T cells in the liver significantly increased after HBV HI compared to mice receiving PBS and Treg depletion further enhanced total CD8+ T cell numbers. A large proportion of the CD8+ T cells that infiltrated the liver after HBV HI became activated as demonstrated by a significant up-regulation of the activation marker CD44, which also indicated antigen-exposure of these cells. The percentage of activated effector CD8+ T cells in the liver of HBV HI mice was further enhanced after Treg depletion (Fig 3B). To quantify the HBV-specific CD8+ T cell response in the liver and spleen of HBV HI mice we utilized the dimer technology. HBV HI resulted in an induction of HBV-specific CD8+ T cells in the liver but not the spleen of mice (Fig 3C). Treg depletion significantly enhanced the percentage of HBV-specific CD8+ T cells among total CD8+ T cells in the liver and spleen. The absolute numbers of HBV-specific CD8+ T cells per organ were also increased significantly after Treg depletion. Liver Tregs also influenced functional properties of CD8+ T cells, as Treg depletion by diphtheria toxin injection significantly increased the percentage of liver CD8+ T cells that expressed the cytotoxic molecule granzyme B (Fig 3D). This functional improvement of CD8+ T cell after Treg depletion was also seen in the spleen of HBV HI mice. It was therefore not surprising to find that the augmented HBV-specific CD8+ T cell responses after Treg depletion correlated with decreased serum HBV DNA levels in HBV HI mice compared to non-depleted animals (Fig 4). Moreover, we found that Treg depletion significantly increased the frequency of CD4+ T cells in the liver but not the spleen of HBV HI mice (S2 Fig). Treg depletion also strongly enhanced CD4+ T cell activation in both the liver and the spleen of HBV HI mice as demonstrated by a significant up-regulation of the activation marker CD43 and down-regulation of CD62L expression on the cells. In comparison, HBV HI alone did not result in significant CD4+ T cell activation as comparable CD43 and CD62L expression levels were observed between HBV HI mice and naive mice (S2 Fig).

Taken together, our results indicate that nTregs infiltrate the liver upon local HBV antigen expression, but do not get activated nor proliferate. However, they are still able to suppress HBV-specific CD8+ T cells responses and hence interfere with viral antigen clearance.

## Discussion

During an infection, a host needs to generate efficient immune responses against the invading pathogen, but exuberant immune responses often lead to immunopathology. Tregs play a very important role in controlling immunopathogenic reactions to infection by dampening pathogen-specific immune responses [17, 45]. However, increased numbers of Treg might also lead to effector to cell dysfunction and subsequent chronicity of the pathogen. In HBV infection, the results of studies determining Treg frequencies in patients with different infection status have been somewhat contradictory. Some studies have reported that the frequency of Tregs in CHB patients was significantly higher than that in healthy controls and those with resolved HBV infection [29, 30, 46] Others did not find difference in Treg frequencies between CHB patients and healthy controls [31, 47, 48]. In our current study, we used the HBV hydrodynamic injection mouse model to study the Treg response in the liver and spleen induced by HBV replication. We could show that HBV replication resulted in a significant increase of the total number of Tregs in the liver but not the spleen compared to control mice. This was in line with results from the FV model, which showed that Treg expansion only occurs in organs of active virus replication [14]. However, no difference was observed in Treg frequencies of total CD4+ T cells indicating that Tregs as well as conventional CD4+ T cell infiltrate the liver after HI of HBV. Further analysis of Treg proliferation by Ki-67 staining also confirmed that there was no difference in frequencies of proliferating Tregs between HBV HI mice and control mice. Although we believed that the increase of CD4+ T cell numbers in the liver is best explained by cell infiltration, we could not completely exclude the possibility that increased survival of liver CD4+ T cells might also contribute for this result. In a previous study in an AdHBV infection mouse model [33], an increase of Treg numbers in the liver was observed as early as 7 days after AdHBV infection. Interestingly, increased Treg frequencies were not observed at this time point but only at day 21 postinfection, suggesting that both Tregs and CD4+ T cells increased in number at day 7. This is very similar to what we found in our model at the later time point after infection. However, we did not find enhanced frequencies of Tregs in the HI model. This could be due to the different viremia kinetics of the two HBV infection mouse models, as HBV viremia lasted for more than 70 days in the AdHBV mouse model but was rapidly cleared within 28 days in the HI mouse model. Our results suggest that absolute numbers of Tregs may be a better marker for evaluating the immune status of HBV patients than the frequency of Tregs. In addition, Treg numbers have to be determined in the liver, as data from blood samples might be misleading.

We previously reported that Treg expansion during FV infection occurs shortly after a massive activation and proliferation of virus-specific effector CD8+ T cells [26]. In the HBV hydrodynamic injection (pSM2) mouse model the massive activation and proliferation of HBV specific CD8+ T cells in liver and spleen was always detected around 21 days post injection (unpublished data). Therefore, we chose day 21 as the time point for our Treg analysis in this study. Differently, in AdHBV mouse model HBV specific CD8+ T cell activation reaches its first peak from day 7 to day 14 [49]. Consistently, Treg analysis was performed at much earlier time points and the increase in Treg numbers peaked also at day 7 postinfection [33].

It has been reported that the HBV-specific immune response was suppressed by Tregs in patients with HBV infection [31]. The suppressive activity of Tregs may be unrelated to the status of HBV infection, because both Tregs from CHB patients and patients with resolved HBV infection could suppress HBV specific CD8+ T cells [31]. This led to the question whether Tregs are specifically activated and differentiated during HBV infection, which had not been studied in previous reports on Tregs in HBV mouse models [33, 50–52]. Helios was identified as an activation marker for Tregs and defines a highly suppressive Treg subset [53–55]. Helios+ Tregs have been shown to exhibit superior suppressive activity than Helios− Tregs in mice [56]. In human Tregs, Helios has been reported to bind the FoxP3 promoter and enhance FoxP3 expression. In turn, Helios knockdown impaired the suppressive activity of Tregs and down regulated FoxP3 expression [57]. TNFR2 is another important marker preferentially expressed by highly activated and functional human and mouse Tregs, and mediates the activating effect of TNFα on Tregs [23, 28]. Tregs that express TNFR2 exhibited a more potent suppressive activity compared to TNFR2− Tregs [58, 59]. We analyzed Helios and TNFR2 expression in liver and spleen Tregs after HBV HI but did not find increased percentages of Tregs expressing theses activation markers. KLRG1 is considered a differentiation marker for Tregs [25]. KLRG1+ Tregs represent a short-lived terminally differentiated Treg subset with recent Ag-encounter and enhanced expression levels of suppressive molecules. However, we also did not observe significant changes in KLRG1+ expression on Tregs in response to HBV replication. Also the two nTreg populations (Vβ5- and Vβ5+ Tregs) that expand after FV infection were not affected after HBV HI in the liver or spleen [26, 28]. Therefore, our results support the conclusion that HBV infection recruits CD4+ T cells to the liver that include nTregs. Although this results in an increase in overall Treg numbers the Tregs do not get activated or differentiated nor proliferate after HBV infection. However, our Treg depletion results and the results from Stross et al. [33], demonstrate that the nTregs that infiltrate the liver after virus infection were still able to suppress HBV-specific T cell responses despite their low activation status. As a consequence, depletion of these cells enhanced HBV-specific CD8+ T cell responses and accelerated viral clearance. Besides, depletion of Tregs also resulted in increased effector CD4+ T cell responses. We could not completely exclude the possibility that increased CD4+ T cell activation might also contribute for the enhanced CD8+ T cell responses.

Taken together, in this study we demonstrated that HBV replication in the liver results in Tregs infiltration into the organ. In contrast to Tregs in other viral infections, these nTregs were not activated or differentiated, but they were still able to suppress HBV-specific CD8+ T cells responses and hence interfered with viral antigen clearance.

## Supporting Information

S1 Fig Gating strategy of Foxp3 expression in CD4+ T cells and phenotype analysis of Tregs by flow cytometry. (DOC)

S2 Fig Impact of Treg depletion on CD4+ T cell responses in HBV-replicating mice. (DOC)
