# Supplementary material for: Characterization of the Treg Response in the Hepatitis B Virus Hydrodynamic Injection Mouse Model
Source: PLoS One. 2016 Mar 17;11(3):e0151717. doi: 10.1371/journal.pone.0151717 (PMC4795771; doi:10.1371/journal.pone.0151717)
Supplement: S1 Fig — (DOC) [file pone.0151717.s001.doc]

**
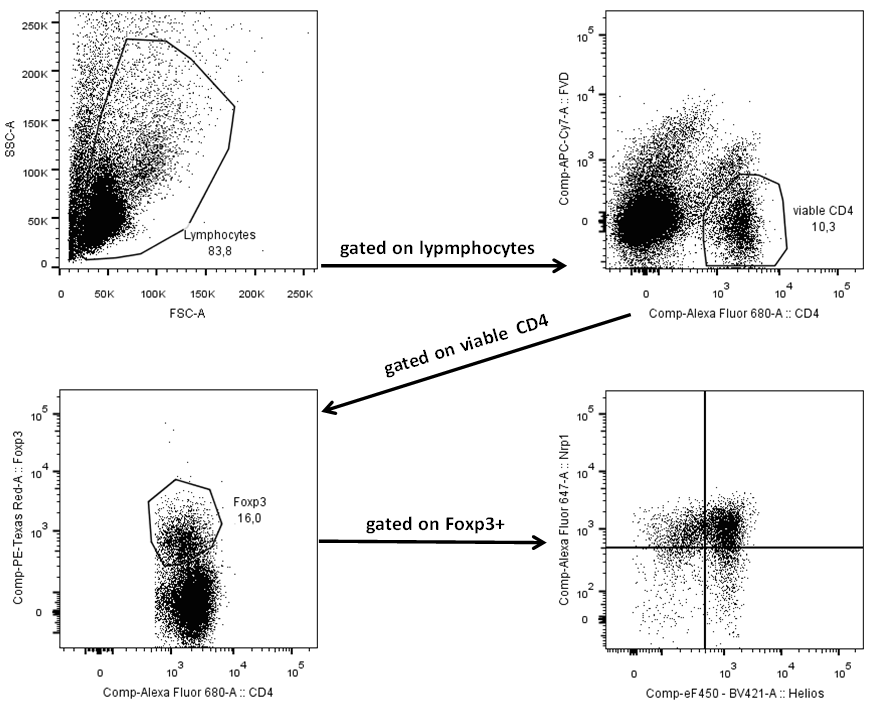
**

**S1 figure. Gating strategy of Foxp3 expression in CD4+ T cells and phenotype analysis of Tregs by flow cytometry.**
