# Supplementary material for: Characterization of the Treg Response in the Hepatitis B Virus Hydrodynamic Injection Mouse Model
Source: PLoS One. 2016 Mar 17;11(3):e0151717. doi: 10.1371/journal.pone.0151717 (PMC4795771; doi:10.1371/journal.pone.0151717)
Supplement: S2 Fig — (DOC) [file pone.0151717.s002.doc]

**
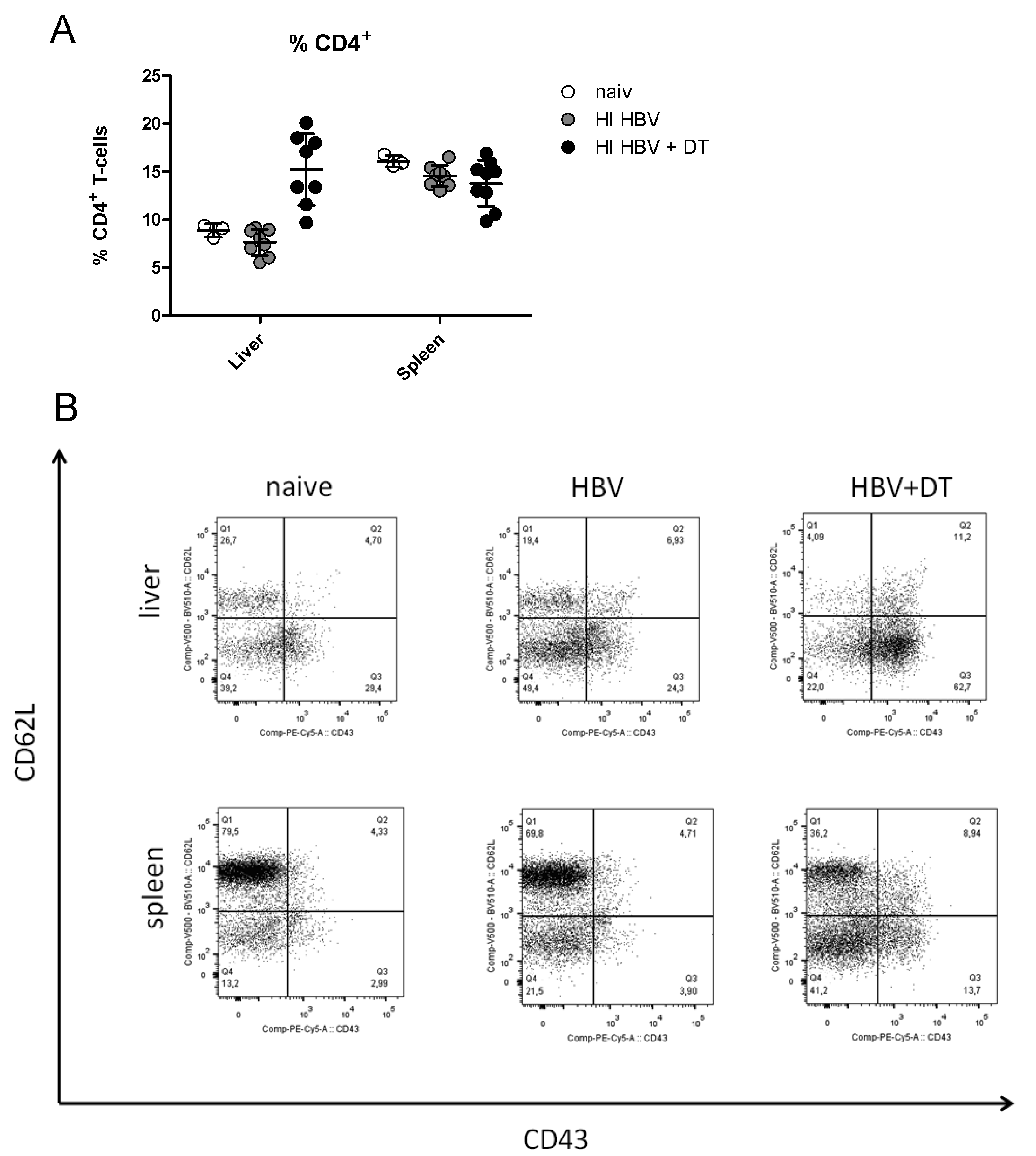
**

**S2 figure. Impact of Treg depletion on CD4+ T cell responses in HBV-replicating mice.** DEREG mice were hydrodynamically injected with pSM2 plasmid and were treated with DT to deplete Foxp3+ Treg on day 10, 12 and 14 after HI. Mice were sacrificed on 21dpi for CD4+ T cell response analysis by flow cytometry. (A) Frequencies of CD4+ T cells were calculated in the liver and spleen. (B) Activation markers CD43 and CD62L expression in intrahepatic and splenic CD4^+^ T cells was measured.
